# Fostering Loyalty and Creativity: How Organizational Culture Shapes Employee Commitment and Innovation in South Korean Firms

**DOI:** 10.3390/bs15040529

**Published:** 2025-04-14

**Authors:** Jiangmin Ding, Gahye Hong

**Affiliations:** 1 BK21 Digital Finance Education and Research Center, College of Business, Pusan National University, Busan 46241, Republic of Korea; djm0823@pusan.ac.kr; 2 College of Business, Pusan National University, Busan 46241, Republic of Korea

**Keywords:** innovation-oriented culture, relationship-oriented culture, organizational commitment, innovative employee behavior, social exchange theory, Human Capital Corporate Panel (HCCP)

## Abstract

Organizational culture, human capital, and innovative capabilities are essential resources for any business, particularly during challenging times. Companies can leverage these resources to gain a competitive advantage. Based on social exchange theory, this study explores the impact of corporate culture on employee commitment and innovative behavior at the individual level, emphasizing the importance of innovation within employee roles. Using 9512 valid data points from the Human Capital Corporate Panel (HCCP), this study validates the research model and hypotheses. The results indicate that an innovation-oriented organizational culture significantly enhances employees’ organizational commitment, which in turn promotes innovative behavior. Organizational commitment is a positive mediating factor in this process. Furthermore, a relationship-oriented culture positively moderates the influence of innovation culture on employees’ organizational commitment. Situated in the Korean context, where Confucian values and collectivism strongly influence workplace dynamics, this study highlights the importance of aligning innovation efforts with cultural expectations. The results suggest that fostering innovative and relational cultural values can be a powerful method in encouraging commitment and creativity, especially in the Korean context companies.

## 1. Introduction

In a dynamic and highly competitive business environment, companies face internal and external challenges in sustaining continuous operations in the market. Janssen (2004) emphasizes that in intensely competitive environments, innovation becomes imperative because it bolsters competitiveness across individual, group, and organizational dimensions. Innovation is the process of deriving economic or social value from knowledge, encompassing the generation, dissemination, and transformation of knowledge into novel or substantially enhanced products or processes, ultimately benefiting society (Raykov, 2014). Innovation capability serves as an intangible asset crucial for a company’s development. Azeem et al. (2021) found that organizational innovative capability helps industrial enterprises in participating in global competition by producing new/improved products and procedures to maintain a sustainable competitive advantage. Furthermore, scholars have confirmed that innovation in business models contributes to improving corporate performance (Bhatti et al., 2021).

In today’s competitive business environment, employee innovation is a critical driver of organizational success. Employees generate value by exploring new processes, developing new products, and establishing creative customer–supplier relationships (Mao & Weathers, 2019). Therefore, understanding employee innovation is critical for a firm’s success (Wallace et al., 2016). However, there remains a relative lack of focus on understanding individual innovation processes. In general, the organizational environment, particularly its culture, affects employees’ innovative behaviors. Organizational culture describes an organization’s unique attributes, expressed through shared beliefs and values established by its founders and conveyed through various means. This shapes employees’ perceptions, behaviors, and principles applicable to organizational members, making them key antecedents of an organization’s performance (Hogan & Coote, 2014; Jigjiddorj et al., 2021). Organizational culture has long been an important topic in management. Among various frameworks, Cameron and Quinn (1999)’s classification has long been widely used in organizational behavior research. They categorized organizational culture into four types: clan, adhocracy, hierarchy, and market culture. Among these, research shows that innovation-oriented (adhocracy) and relationship-oriented (clan) cultures are particularly relevant in fostering innovative behavior, as these cultures promote psychological safety and autonomy atmospheres under which individuals are more likely to perceive innovation (Duygulu & Özeren, 2009; Mehmet, 2021). Accordingly, this study focused on these two cultural dimensions, particularly to examine their impact on employees’ organizational commitment and innovative behavior. An innovation-oriented culture refers to an organizational environment in which employees are often more active and willing to express new ideas and creativity (Malibari & Bajaba, 2022). A relationship-oriented culture, also known as clan culture, emphasizes values such as trust, teamwork, participation, loyalty, and morale (Cameron & Quinn, 1999; Schimmoeller, 2010).

Although the literature suggests that each cultural dimension contributes uniquely to employees’ innovative behaviors, their interactions may be particularly significant depending on the national culture. Although organizational and national cultures are conceptually distinct (Cameron & Quinn, 1999; Hofstede, 1980), they often interact in practice. National culture shapes how organizational values are perceived and enacted by employees. In collectivist contexts like South Korea, clan culture, which emphasizes trust and relational harmony, may align closely with societal norms influenced by Confucian values (Hofstede, 1980). Therefore, even when an organization promotes an innovation-oriented culture, its effectiveness may largely depend on the presence of a strong relationship-oriented culture that reinforces psychological safety and social cohesion. Thus, we argue that the interaction between adhocracy and clan cultures must be considered to better understand innovation related outcomes in the South Korean context.

Furthermore, organizational commitment may play a key mediating role in this relationship. According to the social exchange theory, interpersonal relationships are formed based on individuals’ subjective cost–benefit analysis, leading them to repeat behaviors that have been rewarded in the past (Homans, 1958). In an organizational context, employees’ behaviors are largely influenced by their perception of organizational support and rewards. Parzefall and Salin (2010) found that when employees experience a positive work environment and favorable benefits, they develop a sense of obligation and become more actively engaged in their work. Therefore, organizational commitment can serve as an essential psychological mediator, i.e., employees may develop emotional attachment when they perceive themselves as in alignment with their organization’s culture and values. That is, if employees perceive that the organization fully supports them through an innovation- and relationship-oriented culture, they are likely to develop a higher level of organizational commitment. This heightened commitment motivates employees to engage more actively in innovative behavior, contributing to organizational success. Therefore, this study explored the mechanisms through which organizational culture influences employee innovation in South Korean workplaces. Specifically, we investigated how the interaction between innovation- and relationship-oriented cultures creates an environment that fosters employee innovation. Furthermore, we examined the mediating role of organizational commitment, highlighting how employees’ emotional attachment to their organization bridges the relationship between an innovation-oriented culture and innovative behavior. This study focused on South Korean firms to provide deeper insights into the unique cultural and psychological mechanisms that drive employee innovation in a collectivist and relationally oriented organizational context.

The structure of this paper is as follows. Section 2 elucidates the theoretical background and relevant research and proposes corresponding hypotheses. Section 3 and Section 4 introduce the research methodology, data analysis, and results. In Section 5, we discuss the validation results and conclude.

## 2. Literature Review

### 2.1. Innovative Behavior

Research indicates that employee satisfaction and sustained participation in service innovation are crucial drivers for businesses to gain a competitive advantage (Su & Hahn, 2025). In business operations, innovation often originates from employees’ proactive exploration, rather than solely relying on top-down institutional changes. Therefore, how to enhance employee satisfaction and stimulate their innovative potential has become a widely discussed topic in both academia and industry (Alzghoul et al., 2024).

Tang et al. (2019) point out that employees’ innovative behavior plays a key role in optimizing products, services, processes, and business models. Such behavior not only generates novel and valuable concepts and solutions but also enhances the company’s market adaptability. They further define employee innovation as the creative pursuit, development, implementation, and successful application of new technologies, processes, or products to drive the sustainable development of an organization. Additionally, research shows that innovative work behavior has become a key factor in optimizing product and service processes (Shang et al., 2025). Hock-Doepgen et al. (2025) further confirm that employee innovation behavior not only enhances internal innovation efficiency but also effectively drives business model transformation.

Given the critical role of employee innovation behavior in a company’s competitiveness, academia has extensively explored its influencing factors and mechanisms in recent years. Zhan et al. (2025) demonstrate that in the context of increasing uncertainty in both internal and external environments, psychological safety is considered a crucial foundation for employees to engage in innovative behavior. Ajmal et al. (2025) focus on the hotel service industry and confirm the positive impact of ambidextrous leadership on promoting employee innovation behavior. In contrast, exploitative leadership is negatively correlated with service innovation behavior among hotel employees by lowering employee relational energy (Zhao et al., 2025). In conclusion, employee innovation behavior plays a crucial role in enhancing a company’s competitiveness and market adaptability, making it an important area that businesses must pay close attention to.

### 2.2. Organizational Commitment

Organizational commitment refers to the ability and willingness of employees to align their behaviors with the organization’s needs, goals, and values to maximize organizational benefits (E-Vahdati et al., 2023). McShane and Von Glinow (2003) highlight that the characteristic of organizational commitment is an emotional expression demonstrated by employees, including feelings of belonging, identification, and involvement with the organization. When employees feel proud of their organization, they are often willing to go beyond their daily responsibilities, showing a high degree of responsibility and commitment. Loyal employees are generally more engaged in their work, which in turn enhances overall performance (Allen & Shanock, 2013).

Murray and Holmes (2021) similarly note that employees with strong organizational commitment are more likely to meet or exceed their personal work expectations. This is reflected not only in their positive attitude and efficient execution of tasks but also in their decision-making, where they prioritize actions that align with the organization’s best interests. Such commitment and involvement can help businesses gain a competitive advantage and achieve their goals (Ibrahim et al., 2023).

Therefore, enhancing employee organizational commitment has become an important issue in the field of human resource management. Murray and Holmes (2021) suggest that moderate empowerment fosters the formation of organizational commitment among employees, which in turn helps reduce their turnover. Yasin et al. (2023) verify the positive impact of inclusive leadership and goal alignment on employee organizational commitment. This finding aligns with Lin et al. (2022), who argue that leadership plays a key role in defining organizational culture and cultivating strong employee commitment. On the other hand, the uniqueness of organizational culture determines the direction of the company and the work atmosphere and environment for employees. Culture shapes employees’ cognition and behavior through the dissemination of shared beliefs and values.

However, despite existing research indicating the impact of organizational culture on employee behavior, the specific mechanisms through which cultural characteristics affect employee organizational commitment have not been fully explored. Understanding how different cultural dimensions shape employees’ emotional commitment and behavior is crucial for managers. Therefore, this paper further explores the relationship between organizational culture and employee organizational commitment, providing theoretical insights for the development of effective management strategies.

### 2.3. Organizational Culture

In an organizational context, the shared values, beliefs, and perceptions of employees are referred to as organizational culture (Roy & Mohapatra, 2023). Jigjiddorj et al. (2021) stated that culture delineates the uniqueness of an organization, shaping employees’ perceptions and behaviors through the transmission of common beliefs and values. Perceived organizational culture is defined as employees’ shared values, beliefs, or views about an organization and its environment (Schein, 2010). This uniqueness distinguishes it from other organizations. Azeem et al. (2021) advocated that organizational culture is a crucial input for effective corporate performance, as it determines values, beliefs, and work systems, guiding and providing an appropriate environment for sustainable competition. Furthermore, organizational culture may guide employees in understanding an organization’s core values and foster a shared understanding of organizational processes and goals, thereby encouraging greater involvement.

Organizational culture has long been a central topic in management research. To help organizations understand and optimize their culture for greater efficiency, Cameron and Quinn (1999) classified organizational culture into four types: clan culture, adhocracy culture, hierarchy culture, and market culture. Clan culture, also known as relationship-oriented culture, emphasizes a family-like atmosphere, fostering teamwork, mutual support, and trust (Cameron & Quinn, 1999; Azeem et al., 2021). Employees in such a culture tend to have a strong sense of belonging and organizational identification. Adhocracy culture, also referred to as innovation-oriented culture, prioritizes flexibility and creativity, encouraging risk-taking and experimentation (Cameron & Quinn, 1999; Schimmoeller, 2010). It grants employees greater autonomy and drives continuous innovation and adaptability within the organization. Market culture is results-oriented, focusing on competition, performance, and market success. While it emphasizes customer value and achieving competitive advantages, its highly competitive environment may increase employee stress and weaken emotional commitment (Cameron & Quinn, 1999; Schimmoeller, 2010). Hierarchy culture, on the other hand, values stability, control, and formalization. It features a well-structured organization with centralized decision-making, but its rigid rules and strict hierarchy may suppress innovation and reduce employees’ emotional attachment to the organization (Cameron & Quinn, 1999; Schimmoeller, 2010).

This study focused on clan culture and adhocracy culture, primarily due to their significant impact on organizational commitment and innovative behavior. Clan culture fosters trust and a sense of belonging, strengthening employees’ organizational identification, enhancing loyalty, and reducing turnover (Song et al., 2022). Adhocracy culture promotes flexibility and creativity, encouraging employees to explore new approaches and drive organizational innovation and adaptability. In contrast, market and hierarchy cultures, while emphasizing performance and control, may restrict employee autonomy and creativity, making them less relevant to this study (Richard et al., 2009). Furthermore, employees are more likely to perceive and accept positive organizational cultures. Therefore, this research further explored how clan culture and adhocracy culture influence employees’ organizational commitment and innovative behavior, providing theoretical insights for organizations to develop effective management strategies.

Furthermore, it is important to recognize that organizational culture does not exist in isolation: it is deeply influenced by national culture while also shaping employee behavior and the organizational environment. National culture represents the widely accepted values, behavioral patterns, and beliefs of a society, whereas organizational culture emerges within a specific company, shaping management styles, employee interactions, and workplace dynamics (Steenkamp, 2001; Roy & Mohapatra, 2023), although a distinct national culture often provides the foundational framework for organizational culture, influencing corporate management approaches and employees’ sense of belonging.

South Korea serves as a compelling example. The country’s social culture is heavily influenced by Confucian values, which emphasize hierarchical relationships, collectivism, and respect for authority (Choi et al., 2008; Lee & Kim, 2017). In many traditional South Korean companies, this is reflected in strict hierarchical management and centralized decision-making. However, South Korean culture also places great importance on interpersonal relationships and a sense of belonging, aligning closely with the core principles of clan culture. Many South Korean organizations foster strong workplace relationships, emphasizing teamwork, loyalty, and organizational identification, which in turn strengthen organizational commitment (Kim, 2014). With the rise of the technology sector and entrepreneurial ventures, South Korean companies are increasingly shifting toward an adhocracy culture, which promotes innovation, flexibility, and risk-taking to adapt to rapidly evolving market conditions.

This study focused on South Korea as a research context, incorporating its national cultural background to analyze the impact of organizational culture on employee behavior. By doing so, it provides a more comprehensive understanding of the drivers of organizational commitment and innovation within different cultural frameworks. This approach not only highlights the influence of national culture on organizational culture but also offers valuable theoretical insights for companies seeking to optimize their management strategies across diverse cultural environments.

### 2.4. Theoretical Background

Social exchange theory is a key paradigm for studying organizational behavior. Developed as a sociological theory, social exchange theory examines human social behavior from the perspective of resource exchange. Blau (2017) described social exchanges as voluntary actions by individuals motivated by the rewards they anticipate receiving. Similarly, Homans (1958) posited that interpersonal relationships are formed based on subjective cost–benefit analysis, leading individuals to repeat behaviors that have been rewarded in the past: the more a behavior is rewarded, the more likely it is to be repeated. This theory is commonly applied in organizational behavior research, particularly in the study of organizational support and loyalty perceptions. Parzefall and Salin (2010) found that when employees are presented with a positive work environment and favorable benefits, they feel obliged to engage more actively in their workplace. Conversely, if employees perceive that the organization fails to fulfill its expected obligations, their satisfaction with their work experience decreases (Tekleab et al., 2005). In a relationship-oriented culture, the emphasis on teamwork, shared goals, and supportive relationships aligns closely with the principles of social exchange theory. Employees generate ideas and establish trust-based relationships with organizational members, receiving both social and material rewards. This interaction creates a positive feedback loop, encouraging employees to continue engaging in proactive behaviors, thereby enhancing their commitment to the organization (Kim, 2014). In innovation-oriented cultures, employees are encouraged to generate creative ideas and are rewarded for their contributions. According to social exchange theory, when employees are recognized for their innovative behaviors, they are more likely to actively participate in innovative activities. The relationship between organizational support (such as opportunities for creativity, trust-building relationships, and reward systems) and innovative behavior becomes particularly pronounced when employees perceive that their ideas are valued and when they operate within a culture that supports both personal growth and organizational development (Song et al., 2022). This combination of organizational culture and the principles of social exchange creates an environment in which employees feel that their contributions are reciprocated. Through this positive social exchange loop, employees not only feel material support from the organization but also emotional support, which strengthens their innovation capacity and organizational commitment (Kim, 2014; Song et al., 2022). Therefore, the interplay between organizational culture and social exchange provides crucial boundary conditions for enhancing employees’ organizational commitment and innovative behavior.

### 2.5. Hypothesis Development

An innovation-oriented culture is one in which companies promote innovation without suppressing employees’ creativity and ideas, thereby creating a dynamic and creative work environment for all employees. A company’s main focus is to acquire new resources, produce unique products/services, and enhance its capabilities through novel approaches (Cameron & Quinn, 1999; Schimmoeller, 2010). Such a culture emphasizes flexibility, adaptability, and openness to new ideas, making it a cornerstone of organizational success. Inanlou and Ji-Young (2017) pointed out that an innovation culture highly emphasizes coordination and collaboration among individuals, making employees more proactive in engaging with the organization. Similarly, employees working in an innovative culture often experience higher levels of commitment to the organization (Lok & Crawford, 2001). Vuong et al. (2022)’s research also highlights the crucial role of an innovative climate, specifically emphasizing that an innovative climate strengthens the positive relationship between social support and innovative work behavior.

According to social exchange theory, an innovation-oriented culture provides employees with opportunities to contribute creatively and receive recognition and support in return. Organizations and leaders who create a supportive atmosphere leave a positive social exchange impression on employees. Consequently, this leads employees to develop a sense of obligation and reciprocate with loyalty to the organization. Such reciprocal relationships strengthen employees’ commitment to the organization, as they perceive an innovation-oriented culture as both rewarding and aligned with their professional aspirations.

**H1.**  *Innovation-oriented culture is positively related to organizational commitment*.

A relationship-oriented culture emphasizes values such as trust, teamwork, participation, loyalty, and morale (Cameron & Quinn, 1999; Schimmoeller, 2010), creating a warm and supportive work environment for employees. This culture encourages companies to focus on developing talent and fostering team spirit, allowing employees to collaborate like a family and feel a strong sense of belonging (Azeem et al., 2021). In the context of South Korean workplaces, in which Confucianism is deeply rooted, organizations tend to favor collectivism over individualism. Relationship-oriented culture aligns closely with collectivist values, making it widely accepted and effective in South Korean organizations. Choi et al. (2008) found that clan culture significantly affects overall job satisfaction, satisfaction with colleagues, supervision, and personal growth. Similarly, Kim (2014) emphasized the importance of transformational leaders in creating a unique clan culture that strengthens the emotional bond between followers and the organization.

A relationship-oriented culture not only enhances employees’ cohesion and trust but also amplifies their perception of an innovation-oriented culture, thereby promoting organizational commitment. This synergy is particularly relevant in South Korea, where relational values, such as harmony and respect, play important roles in the workplace. South Korean organizations are often characterized by hierarchical structures and collectivist values. In such conditions, employees without a strong sense of relational security may be reluctant to share new ideas or take risks. A relationship-based culture addresses such problems by encouraging a supportive and trust-based atmosphere that enables employees to feel safe and confident about generating innovative ideas and taking risks. Therefore, a relationship-oriented culture strengthens the effectiveness of an innovation-oriented culture by providing the trust and support necessary for employees to engage fully in innovative activities. Although an innovation-oriented culture encourages employees to think creatively and propose new ideas, its impact may be limited in the absence of a supportive relational environment. Consequently, in workplaces with a strong relationship-oriented culture, employees are more likely to feel secure and connected to the organization, enabling them to embrace the creative opportunities offered by an innovation-oriented culture without fear of failure or negative consequences. Empirical findings support this. For instance, Lee and Kim (2017) demonstrate that clan culture positively moderates the relationship between corporate social responsibility and company performance among South Korean employees. Yustrilia et al. (2022) found that relationship-oriented culture moderates the relationship between compensation and employee engagement. Based on the above argument, this study suggests that the interaction of relationship-oriented and innovation-oriented approaches jointly encourages a synergy that drives organizational commitment, leading to innovative behavior. Employees who are fully supported by a relational culture are more likely to embrace the opportunities presented by an innovation-oriented culture.

**H2.**  *The positive effect of innovation-oriented culture on organizational commitment is stronger when relationship-oriented culture is high.*

McShane and Von Glinow (2003) indicated that organizational commitment is characterized as an emotional expression exhibited by employees, encompassing feelings of belonging, identification, and involvement. When employees take pride in working for an organization, they can go beyond their daily duties and demonstrate commitment, and loyal employees typically perform better, leading to improved performance (Allen & Shanock, 2013). Fauziawati (2021) confirmed through survey research that organizational commitment significantly improves employees’ innovative work behavior and that organizational commitment can also reduce the negative impact of job insecurity on innovative work behavior. Additionally, Anagha and Magesh (2016) surveyed 483 software engineers in India and found that organizational commitment significantly improves innovative work behavior. Furthermore, Muhamad et al. (2023) emphasize that innovative work behavior and organizational commitment can create successful business performance, such as increased sales, profits, satisfaction, market share, high productivity, employee loyalty, and low employee turnover. Therefore, we propose the following hypothesis:

**H3.**  *Organizational commitment is positively related to innovative behavior.*

Furthermore, this study predicted that employees’ organizational commitment positively mediates the relationship between perceived organizational culture and innovative behavior. Social exchange theory states that a subjective cost–benefit analysis is the foundation for forming interpersonal relationships. Consequently, people often repeat actions that have previously resulted in reward. According to Homans (1958), behaviors are highly likely to be repeated when they are frequently rewarded. Companies with innovation-oriented cultures tend to encourage and incentivize innovation among employees, who are motivated to produce more. Specifically, after entering an organization and identifying and evaluating its culture and values, employees decide on their alignment with the organization, leading to the development of organizational commitment and stimulating their contributions to the organization, including offering new ideas and creativity. However, the relationship between perceived organizational culture and innovative behavior is often mediated by organizational commitment.

Organizational commitment may play a critical mediating role in the South Korean context, where collectivism and relational values heavily influence workplace behaviors. In South Korea, employees tend to place a high value on harmony and loyalty in organizations, reflecting Confucian values. Thus, when employees perceive their organization as supportive through innovative and relational-based cultures, they are more likely to develop a sense of emotional attachment and loyalty toward the organization, which is termed organizational commitment. These heightened attachments toward the organization may lead employees to engage in creative behaviors and take risks beyond their formal roles. Moreover, in South Korean hierarchical organizational culture, employees often hesitate to engage in innovative behavior unless they feel valued and secure. Under such conditions, organizational commitment reflects the psychological reward that employees may perceive as a result of their relationship with the organization, reinforcing their sense of trust and belonging. This sense of commitment serves as a psychological buffer, reducing fear of failure and encouraging employees to engage more actively in innovative behavior. Without such perceived rewards, an innovation-oriented culture may remain ineffective, as employees hesitate to act on their creative potential in the absence of relational and emotional support.

Anagha and Magesh (2016) found that organizational commitment plays a partial mediating role in the relationship between innovation climate, knowledge management, and employees’ innovation motivation. Additionally, the mediating role of organizational commitment has been validated in other contexts. Sharma et al. (2021) found that commitment moderates the relationship between innovation and green performance. Furthermore, Almaaitah et al. (2020) conducted a survey of 385 Jordanian hotel employees and found that effective continuance and normative commitment can moderate the impact of talent management on organizational performance. Hence, the following hypothesis is proposed:

**H4.**  *Organizational commitment mediates the relationship between innovation-oriented culture and innovative employee behavior.*

**Figure 1:**
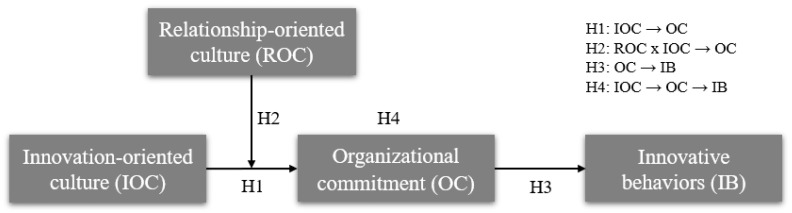
Research model.

Based on this, we constructed the research model shown in Figure 1.

## 3. Research Method

This study examined the relationships among organizational culture, organizational support, and innovative employee behavior in South Korean firms. Building upon previous research, we developed corresponding hypotheses and empirically tested them using a quantitative approach.

Specifically, this study utilized official survey data from South Korea’s Human Capital Corporate Panel (HCCP). We first selected samples that fit the research scope and then organized and summarized the data to ensure the representativeness of the research subjects and the reliability of the data. Subsequently, we measured the study variables based on the literature, ensuring the scientific rigor and comparability of variable definitions and measurement methods. To further validate the relationships among these variables, we applied structural equation modeling (SEM) for analysis.

In the following sections, we provide a detailed explanation of the sample selection and data collection process, elaborate on the variable measurement strategy, and outline the data analysis methods, ensuring transparency and reproducibility in the research design.

### 3.1. Sample and Data Collection

This study employed quantitative analysis to examine the relationships between various variables. Specifically, we utilized data from the Human Capital Corporate Panel (HCCP) survey, conducted jointly by the Korea Research Institute for Vocational Education and Training (KRIVET) and the Ministry of Employment and Labor of South Korea. This dataset is official national statistical data, approved by the National Statistical Office (approval 389003), and provides comprehensive information on human resource management, employee training and skill development, R&D investment, and other key aspects at the enterprise level.

One of the key advantages of the HCCP dataset is its ability to provide comprehensive data on human resource management, as the survey is conducted annually across a wide range of firms. Additionally, since the respondents are employees of the sampled companies, the dataset enables aggregated analysis of employees’ perceptions at the organizational level. However, as the responses are collected at the individual level, the analysis is based on employees’ perceived organizational culture, not on aggregated firm-level constructs.

We conducted our analysis using 9512 samples provided by the HCCP dataset. Table 1 presents the characteristics of the respondents. Overall, the proportion of male respondents (69.82%) was significantly higher than that of female respondents (30.18%). The majority of respondents (80.63%) fell within the 26- to 49-year age group. The HCCP survey targeted companies with more than 100 employees, among which 59.65% had 100–299 employees, 31.29% had 300–999 employees, and only 9.06% had 1000 or more employees. Based the official HCCP classification, the surveyed companies were categorized into three major industries: manufacturing, finance, and non-financial industries. Manufacturing companies accounted for the largest share (77.47%), while finance and non-financial industries made up 5.70% and 16.83%, respectively. Additionally, the distribution of respondents based on tenure at their companies was relatively balanced.

### 3.2. Variable Measurement

Based on the research model depicted in Figure 1, the independent variable in this study was innovation-oriented culture (IOC), with relationship-oriented culture (ROC) serving as the moderating variable, organizational commitment (OC) as the mediating variable, and innovative employee behavior (IB) as the dependent variable. Specifically, IOC is defined as one in which organizational members are proactive, creative, and challenging, emphasizing flexible responses to change by creatively solving problems (Song et al., 2022). ROC emphasizes values such as trust, teamwork, participation, and loyalty (Parker & Bradley, 2000), creating a warm and mutually supportive work environment for employees. Employee OC is described as an emotional manifestation of employees, denoting an individual’s feelings of belonging, identification, and engagement with the organization (McShane & Von Glinow, 2003). IB encompasses the comprehensive performance of employees who creatively seek, establish, implement, and successfully realize new technologies, processes, or products that generate useful products or services (Tang et al., 2019). Based on the questionnaire provided by the HCCP, we compiled and summarized the measurement items for each variable, as detailed in Table 2.

We used SmartPLS 4 software to conduct confirmatory factor analysis and structural equation modeling (SEM) to test the research hypotheses and present the relevant results. SEM is a powerful statistical method used to assess and test causal relationships between latent variables. By using SEM, we can comprehensively analyze the interactions and influence paths between multiple variables, revealing the complex relationships among them (Lam et al., 2021). Specifically, this study employed partial least squares (PLS)-SEM, a method particularly effective in handling complex models, especially when multiple simultaneous relationships exist between variables. This method is widely used in business management research, as it provides accurate and stable estimates when analyzing models with multiple latent variables and complex interaction effects (Hair et al., 2011). Therefore, PLS-SEM served as a robust tool for validating and interpreting the relationships among the variables in this study.

## 4. Data Analysis and Results

First, we analyzed the reliability of each variable. As shown in Table 2, the α and CR values of the four variables were all greater than 0.6, confirming the reliability and validity of the questionnaire for each variable (Nunnally & Bernstein, 1994). The validity of the research instrument was evaluated using convergent and discriminant validity tests. Convergent validity gauges the consistency among variables measuring each latent variable, whereas discriminant validity assesses the independence of measurement variables from other latent variables. As demonstrated in Table 2 and Table 3, the standardized estimates of the measurement variables for each latent variable exceeded 0.5, thus validating convergent validity. Moreover, the square root of AVE surpassed 0.7 and exceeded correlations with other latent variables, ensuring discriminant validity (Barclay et al., 1995; Chin, 1998).

Moreover, we calculated the heterotrait–monotrait (HTMT) ratio to assess discriminant validity. This ratio measures the average correlation of indicators across different constructs relative to the average correlation of indicators measuring the same construct. As shown in Table 4, all HTMT values calculated in this study were below 0.85, indicating a high level of discriminant validity among the variables (Kline, 1998).

**Figure 2:**
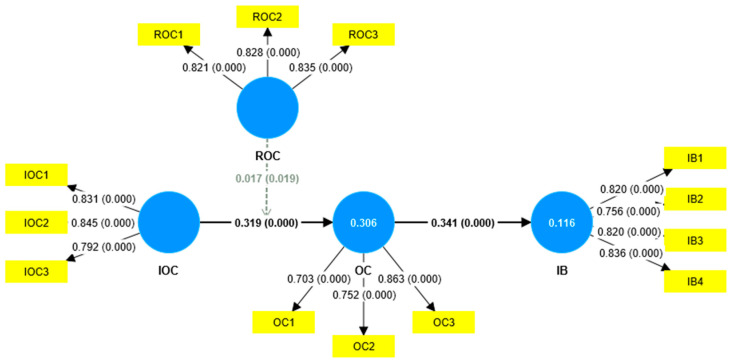
Results of hypothesis testing.

After confirming convergent and discriminant validity, the next step was to test the research hypotheses. The predictive power of a structural model is typically assessed using the R^2^ values of endogenous constructs. The R^2^ values for organizational commitment and innovative behavior were 0.306 and 0.116 (Figure 2), respectively, both meeting the threshold of R^2^ ≥ 0.1, suggesting that the model demonstrated a certain level of predictive capability.

Additionally, model fit was also evaluated using SRMR and NFI in SmartPLS. The SRMR (standardized root mean square residual) measures the discrepancy between the observed correlation matrix and the model-implied correlation matrix, with values below 0.10 or 0.08 generally indicating a good model fit (Hu & Bentler, 1998). The NFI (normed fit index) is calculated as 1 minus the ratio of the chi-squared value of the proposed model to that of the null model, with values ranging from 0 to 1, where higher values indicate better model fit (Lohmöller, 2013). In this study, the SRMR value was 0.066 and the NFI value was 0.81, confirming that the model achieved a satisfactory fit.

In this study, the hypotheses were verified through path analysis, as shown in Figure 2 and Table 5. H1 predicted that an innovation-oriented organizational culture has a positive impact on employees’ organizational commitment. In the results, t = 25.877, *p* < 0.001. Therefore, H1 was supported. Similarly, the results of the validation of H2 showed t = 2.35, *p* < 0.05; thus, H2 was supported. Furthermore, the validation result for H3 showed that t = 34.119, *p* < 0.001, thus supporting H3. This indicates that employees’ organizational commitment can promote innovative behavior. Finally, H4 predicted that organizational commitment positively mediates the relationship between innovation-oriented culture and innovative behavior. The results showed that t = 19.296, *p* < 0.001. Therefore, H4 was supported.

## 5. Discussion and Conclusions

### 5.1. Discussion

Human capital is a critical organizational asset for all companies. Mao and Weathers (2019) noted that employees can create significant value by exploring new processes, inventing new products, and establishing customer–supplier relationships. In highly competitive business environments, employee innovation often becomes a key factor for companies to gain a competitive advantage. Therefore, this study focused on the individual process of employee innovation and constructed a model of the factors influencing innovative employee behavior based on social exchange theory. The study explored the relationship between innovation-oriented culture, organizational commitment, and employee innovation behavior. By analyzing official HCCP data, we found that innovation-oriented culture not only directly promotes employees’ organizational commitment but also indirectly influences their innovative behavior through organizational commitment. This result is consistent with the literature, indicating that organizational culture, especially innovation-oriented culture, shapes employees’ values, behaviors, and sense of identity, thereby enhancing their innovation capacity (Lok & Crawford, 2001; Azeem et al., 2021).

First, innovation-oriented culture improves employees’ sense of identity and belonging to the organization by creating a dynamic, creative, and collaborative work environment (Parker & Bradley, 2000). This finding aligns with the social exchange theory, which suggests that when employees perceive support and incentives from the organization, this strengthens their sense of belonging and resonates with the organization’s values and goals, thus increasing their organizational commitment. In the context of South Korean culture, the moderating role of relationship-oriented culture further strengthens the relationship between innovation-oriented culture and employees’ organizational commitment. Specifically, values such as trust, cooperation, and solidarity create a more stable employee–organization relationship in South Korean companies, positively stimulating innovative behavior. Existing research often focuses on leadership (e.g., Ajmal et al., 2025; Yasin et al., 2023) and other factors influencing employee organizational commitment and innovation behavior. However, it is important to note that the uniqueness of organizational culture largely determines the direction of the organization and the work atmosphere and environment. Culture shapes employees’ cognition and behavior through the dissemination of shared beliefs and values (Azeem et al., 2021). Therefore, the profound impact of organizational culture on employee behavior deserves more attention. Additionally, research indicates that significant cultural differences between countries make the failure to account for these differences a common reason for business failures (Steenkamp, 2001). Organizational culture is largely influenced by national culture, and many existing theories are derived from research in Western countries, particularly the United States. Therefore, it is crucial to examine the applicability of these theories and models in other cultural contexts to identify their universality and boundary conditions (Steenkamp, 2001). This study examined human resource management theory within the specific cultural context of South Korea, which holds significant practical implications.

Furthermore, the results confirmed that employees’ organizational commitment positively influences innovative behavior. This finding is consistent with the results of studies by Fauziawati (2021) and others. Allen and Shanock (2013) emphasize that when employees take pride in working for the organization, they often go beyond their daily duties, demonstrating commitment. Loyal employees typically contribute to better performance, underscoring the significance of organizational commitment in fostering innovation. This finding provides a new perspective on how employees exhibit higher levels of innovative behavior within the organization. On this basis, loyal employees are not only willing to invest more time and effort in their daily tasks but also demonstrate a stronger sense of identification with the organization’s goals. This sense of identification and responsibility further motivates them to come up with new ideas and solutions in their work. Therefore, organizational commitment undoubtedly plays a key role in stimulating employee innovation behavior.

Finally, the research findings confirm that employees’ organizational commitment plays a positive mediating role between innovation-oriented culture and innovation behavior. This finding is similar to that of Anagha and Magesh (2016), who found that organizational commitment mediates the relationship between innovation climate, knowledge management, and employee innovation motivation. According to social exchange theory, employees who perceive and are motivated by the organization’s culture will have more identification and motivation with the organization, which will lead to more innovative ideas and behaviors. In addition, it is important to recognize that organizational commitment plays a crucial role in promoting innovation; however, this relationship may vary across different cultural contexts. Therefore, future cross-cultural studies are needed to validate and refine the generalizability of these findings.

### 5.2. Conclusions

In a highly competitive business environment, employee innovation often becomes a key factor in gaining a competitive advantage for companies. Therefore, this study focused on the individual process of employee innovation, and based on social exchange theory, we constructed a model of factors influencing innovative employee behavior, exploring the relationship between innovation-oriented culture, organizational commitment, and innovative employee behavior. The research findings reveal that innovation-oriented culture not only directly promotes organizational commitment among employees but also indirectly influences their innovative behavior through organizational commitment. In the specific cultural context of South Korea, a relationship-oriented culture positively moderates the impact of an innovation-oriented culture on organizational commitment.

At the same time, Steenkamp (2001) points out that many existing theories originate from Western countries, particularly the United States. Given the evolving nature of these theories and the changing human resource environments, it is important to validate the applicability of these theories and models in other cultural contexts. Additionally, the official HCCP data used in this study accurately reflect the human resource management status at the organizational level. Since the data are derived from employees, aggregating them provides a deeper understanding of employees’ perceptions at the organizational level, thereby enhancing the objectivity and accuracy of the research.

Based on the above findings, this study makes several contributions in the following areas. First, from an academic perspective, this study confirms the critical role of innovation capabilities within organizations and examines the factors influencing the employee innovation process at the individual level. Second, this research extends the application of social exchange theory to the study of innovative employee behavior, not only reaffirming the theory but also enriching the related research. Third, the study’s results validate the significant influence of both innovation culture and relationship-oriented culture, which aligns with the findings of Azeem et al. (2021). The combination of organizational culture and employee behavior provides valuable insights for future research. Furthermore, Steenkamp (2001) pointed out that neglecting cultural differences is often a reason for the failure of many companies. In response to this, this study further explored organizational culture within the specific cultural context of South Korea, refining the research on human resources within this cultural background.

From a practical perspective, the findings of this study provide important insights for business management, particularly in the context of South Korean culture. First, an innovation-oriented culture and relationship-oriented culture are key factors in enhancing organizational commitment within a company. In South Korean enterprises, a relationship-oriented culture emphasizes values such as trust, cooperation, and unity, which help strengthen the emotional bond between employees and the organization. The emphasis on collectivism and social harmony in South Korean culture makes it easier for employees to develop a sense of identification in an environment with shared values. Therefore, companies should focus on fostering an innovative, harmonious, and collaborative cultural atmosphere to enhance employees’ sense of belonging and alignment with the organization, thereby stimulating their innovation motivation and increasing engagement. The findings should be interpreted in light of the national cultural context in which the study was conducted. South Korea’s collectivist orientation and Confucian heritage emphasize relational harmony, loyalty, and respect for hierarchy. These cultural characteristics likely reinforced the salience of clan culture and shaped how employees responded to organizational cues. In particular, the interaction effect observed between innovation- and relationship-oriented cultures may reflect the cultural expectation that change and creativity occur within a foundation of trust and shared values. Thus, rather than functioning independently, innovation-oriented culture may exert stronger effects in environments where relational norms are institutionally embedded. This suggests that the effectiveness of organizational culture types is contingent upon broader societal values, and interpretations of culture–behavior relationships must consider such contextual influences.

Second, this study confirms the positive impact of organizational commitment on employee innovation behavior. As a result, companies should prioritize strengthening the emotional connection between employees and the organization, not only by using incentive measures to drive innovation but also by improving organizational culture to enhance employees’ loyalty and commitment. By reinforcing the role of organizational values as a bond, companies can better inspire innovative ideas from employees, boosting the organization’s competitiveness. Finally, this study examines the innovation process from an individual perspective, providing valuable insights for companies to improve their human resource management practices. In South Korean culture, employees tend to demonstrate higher levels of innovation in stable, trust-filled work environments. Therefore, companies should focus on addressing employees’ psychological needs and creating a more supportive and inclusive work environment to tap into their innovation potential.

In conclusion, considering the cultural context of South Korea, companies implementing innovation-driven strategies must pay special attention to the influence of cultural factors, particularly how innovation-oriented and relationship-oriented cultures promote organizational commitment and innovation behavior. By strengthening cultural development, companies can foster employees’ innovation awareness and enhance the long-term competitiveness of the organization.

The study has several limitations. First, this research focuses on organizational behavior within the specific cultural context of South Korea. Although South Korea has unique cultural characteristics, cultural differences across countries and regions may influence employees’ innovation behavior and organizational commitment. Therefore, cross-cultural comparative research is crucial for testing the applicability and generalizability of the findings in other cultural contexts. Second, this study relies on self-reported survey data from employees, which may be subject to social desirability bias. Future research could consider using multiple data collection methods, such as interviews, observations, or experimental designs, to reduce the impact of self-report bias. Finally, although this study uses social exchange theory to explain employees’ innovation behavior, the driving factors of innovation behavior may be more diverse. Other theoretical frameworks, such as motivation theory and social identity theory, could provide different perspectives for explaining employee innovation behavior. Therefore, future research could combine multiple theories to construct a more comprehensive framework and explore the influencing factors of innovation behavior in depth. Overall, the development of businesses is inseparable from employee participation, and employee innovation behavior is considered a competitive advantage for companies. Therefore, research on employee psychology and behavior should continue to deepen, exploring more dimensions of influencing factors and mechanisms behind innovation behavior.

## Figures and Tables

**Table 1:** Characteristics of respondents (N = 9512).

Category	Items	Frequency	Percentage (%)
Gender	Male	6641	69.82%
	Female	2871	30.18%
Age	0–25	113	1.19%
	26–49	7670	80.63%
	50 and above	1729	18.18%
Educational background	High school graduate or less	995	10.46%
	College	2277	23.94%
	Bachelor’s degree	5705	59.98%
	Master’s degree	499	5.25%
	PhD	36	0.37%
Firm size	100–299	5674	59.65%
	300–999	2976	31.29%
	1000 and above	862	9.06%
Industry type	Manufacturing industry	7369	77.47%
	Financial industry	542	5.70%
	Non-financial industry	1601	16.83%
Years of work	Less than 5 years	2524	26.53%
	5–10 years (including 5 years)	2646	27.82%
	10–15 years (including 10 years)	1925	20.24%
	15–20 years (including 15 years)	1068	11.23%
	20 years and above	1349	14.18%

**Table 2:** Variable measurement, reliability, and convergent validity analysis.

Variables and Measurement	Loading
Innovation-oriented culture (α = 0.762, C.R. = 0.766, AVE = 0.677).	
IOC1. Our company encourages change and trying new things.	0.831
IOC2. Our company rewards innovation appropriately.	0.845
IOC3. Our organization favors creative people over conscientious people.	0.792
Relationship-oriented culture (α = 0.771, C.R. = 0.771, AVE = 0.686).	
ROC1. Our company has a family-like organizational atmosphere.	0.821
ROC2. Our company emphasizes unity and togetherness.	0.828
ROC3. Our company emphasizes teamwork.	0.835
Organizational commitment (α = 0.672, C.R. = 0.716, AVE = 0.601).	
OC1. I feel the problems of this company as if they were my own.	0.703
OC2. If I decide to leave this company, I will lose too much in my life.	0.752
OC3. This company is worthy of my loyalty.	0.863
Innovative behavior (α = 0.824, C.R. = 0.833, AVE = 0.654).	
IB1. Ability to develop new products (goods/services)	0.820
IB2. Efficiency and simplification of business processes	0.756
IB3. Securing competitive advantage by reducing the cost of products (goods/services)	0.820
IB4. Quality of products (goods/services)	0.836

**Table 3:** Fornell–Larcker criterion.

	OC	IB	IOC	ROC
OC	0.775			
IB	0.341	0.809		
IOC	0.498	0.429	0.823	
ROC	0.490	0.412	0.599	0.828

**Table 4:** Heterotrait–monotrait ratio.

	OC	IB	IOC	ROC
OC				
IB	0.442			
IOC	0.677	0.534		
ROC	0.663	0.514	0.780	

**Table 5:** Hypothesis testing and results.

Hypothesis	Path	Path Coefficient	T Values	Results
H1	IOC -> OC	0.319	25.877 ***	Supported
H2	ROC × IOC -> OC	0.017	2.35 *	Supported
H3	OC -> IB	0.341	34.119 ***	Supported
H4	IOC -> OC -> IB	0.109	19.296 ***	Supported

Notes: * *p* < 0.05, *** *p* < 0.001.

## Data Availability

The original contributions presented in the study are included in the article. Further inquiries can be directed to the corresponding author.

## References

[B1-behavsci-15-00529] Ajmal M., Sareet Z., Islam A. (2025). Unleashing innovation through employee voice behavior in the hotel industry: The impact of ambidextrous leadership on innovative work behavior. Journal of Hospitality and Tourism Insights.

[B2-behavsci-15-00529] Allen D. G., Shanock L. R. (2013). Perceived organizational support and embeddedness as key mechanisms connecting socialization tactics to commitment and turnover among new employees. Journal of Organizational Behavior.

[B3-behavsci-15-00529] Almaaitah M., Alsafadi Y., Altahat S., Yousfi A. (2020). The effect of talent management on organizational performance improvement: The mediating role of organizational commitment. Management Science Letters.

[B4-behavsci-15-00529] Alzghoul A., Khaddam A. A., Alshaar Q., Irtaimeh H. J. (2024). Impact of knowledge-oriented leadership on innovative behavior, and employee satisfaction: The mediating role of knowledge-centered culture for sustainable workplace. Business Strategy & Development.

[B5-behavsci-15-00529] Anagha K., Magesh R. (2016). Motivation to innovate: An examination of the role of employee commitment. Asian Journal of Research in Social Sciences and Humanities.

[B6-behavsci-15-00529] Azeem M., Ahmed M., Haider S., Sajjad M. (2021). Expanding competitive advantage through organizational culture, knowledge sharing and organizational innovation. Technology in Society.

[B7-behavsci-15-00529] Barclay D., Higgins C., Thompson R. (1995). The partial least squares (PLS) approach to casual modeling: Personal computer adoption and use as an Illustration. Technology Studies.

[B8-behavsci-15-00529] Bhatti S. H., Santoro G., Khan J., Rizzato F. (2021). Antecedents and consequences of business model innovation in the IT industry. Journal of Business Research.

[B9-behavsci-15-00529] Blau P. (2017). Exchange and power in social life.

[B10-behavsci-15-00529] Cameron K. S., Quinn R. E. (1999). Diagnosing and changing organizational culture: Based on the competing values framework.

[B11-behavsci-15-00529] Chin W. W. (1998). The partial least squares approach to structural equation modeling. Modern Methods for Business Research.

[B12-behavsci-15-00529] Choi Y. S., Martin J. J., Park M. (2008). Organizational culture and job satisfaction in Korean professional baseball organizations. International Journal of Applied Sports Sciences.

[B13-behavsci-15-00529] Duygulu E., Özeren E. (2009). The effects of leadership styles and organizational culture on firm’s innovativeness. African Journal of Business Management.

[B14-behavsci-15-00529] E-Vahdati S., Noor N. A. M., Mah P. Y., Chuah F., Md Isa F. (2023). Social and environmental sustainability, workers’ well-being, and affective organizational commitment in palm oil industries. Sustainability.

[B15-behavsci-15-00529] Fauziawati D. (2021). The effect of job insecurity on innovative work behavior through organizational commitment in UFO elektronika employees. Journal of Business and Management Review.

[B16-behavsci-15-00529] Hair J. F., Ringle C. M., Sarstedt M. (2011). PLS-SEM: Indeed a silver bullet. Journal of Marketing theory and Practice.

[B17-behavsci-15-00529] Hock-Doepgen M., Montasser J. S., Klein S., Clauss T., Maalaoui A. (2025). The role of innovative work behavior and organizational support for business model innovation. R&D Management.

[B18-behavsci-15-00529] Hofstede G. (1980). Culture and organizations. International Studies of Management & Organization.

[B19-behavsci-15-00529] Hogan S. J., Coote L. V. (2014). Organizational culture, innovation, and performance: A test of Schein’s model. Journal of Business Research.

[B20-behavsci-15-00529] Homans G. C. (1958). Social behavior as exchange. American Journal of Sociology.

[B21-behavsci-15-00529] Hu L. T., Bentler P. M. (1998). Fit indices in covariance structure modeling: Sensitivity to underparameterized model misspecification. Psychological Methods.

[B22-behavsci-15-00529] Ibrahim B., Zumrah A. R., Supardi S., Juhji J. (2023). Transformational leadership and organizational commitment: Moderator role of pesantren employee job satisfaction. International Journal of Evaluation and Research in Education.

[B23-behavsci-15-00529] Inanlou Z., Ji-Young A. (2017). The effect of organizational culture on employee commitment: A mediating role of human resource development in Korean firms. Journal of Applied Business Research.

[B24-behavsci-15-00529] Janssen O. (2004). How fairness perceptions make innovative behavior more or less stressful. Journal of Organizational Behavior.

[B25-behavsci-15-00529] Jigjiddorj S., Zanabazar A., Jambal T., Semjid B. (2021). Relationship between organizational culture, employee satisfaction and organizational commitment. SHS web of conferences.

[B26-behavsci-15-00529] Kim H. (2014). Transformational leadership, organizational clan culture, organizational affective commitment, and organizational citizenship behavior: A case of South Korea’s public sector. Public Organization Review.

[B27-behavsci-15-00529] Kline R. B. (1998). Software review: Software programs for structural equation modeling: Amos, EQS, and LISREL. Journal of Psychoeducational Assessment.

[B28-behavsci-15-00529] Lam L., Nguyen P., Le N., Tran K. (2021). The relation among organizational culture, knowledge management, and innovation capability: Its implication for open innovation. Journal of Open Innovation: Technology, Market, and Complexity.

[B29-behavsci-15-00529] Lee M., Kim H. (2017). Exploring the organizational culture’s moderating role of effects of Corporate Social Responsibility (CSR) on firm performance: Focused on corporate contributions in Korea. Sustainability.

[B30-behavsci-15-00529] Lin M., Zhang X., Ng B. C. S., Zhong L. (2022). The dual influences of team cooperative and competitive orientations on the relationship between empowering leadership and team innovative behaviors. International Journal of Hospitality Management.

[B31-behavsci-15-00529] Lohmöller J. B. (2013). Latent variable path modeling with partial least squares.

[B32-behavsci-15-00529] Lok P., Crawford J. (2001). Antecedents of organizational commitment and the mediating role of job satisfaction. Journal of Managerial Psychology.

[B33-behavsci-15-00529] Malibari M. A., Bajaba S. (2022). Entrepreneurial leadership and employees’ innovative behavior: A sequential mediation analysis of innovation climate and employees’ intellectual agility. Journal of Innovation & Knowledge.

[B34-behavsci-15-00529] Mao C. X., Weathers J. (2019). Employee treatment and firm innovation. Journal of Business Finance & Accounting.

[B35-behavsci-15-00529] McShane S. L., Von Glinow M. A. (2003). Organizational behavior.

[B36-behavsci-15-00529] Mehmet S. (2021). Effect of organizational culture on innovation in Finnish companies. Master’s Thesis.

[B37-behavsci-15-00529] Muhamad L. F., Bakti R., Febriyantoro M. T., Kraugusteeliana K., Ausat A. M. A. (2023). Do innovative work behavior and organizational commitment create business performance: A literature review. Community Development Journal: Jurnal Pengabdian Masyarakat.

[B38-behavsci-15-00529] Murray W. C., Holmes M. R. (2021). Impacts of employee empowerment and organizational commitment on workforce sustainability. Sustainability.

[B39-behavsci-15-00529] Nunnally J., Bernstein I. (1994). Psychometric theory.

[B40-behavsci-15-00529] Parker R., Bradley L. (2000). Organisational culture in the public sector: Evidence from six organisations. International Journal of Public Sector Management.

[B41-behavsci-15-00529] Parzefall M. R., Salin D. M. (2010). Perceptions of and reactions to workplace bullying: A social exchange perspective. Human Relations.

[B42-behavsci-15-00529] Raykov M. (2014). Employer support for innovative work and employees’ job satisfaction and job-related stress. Journal of Occupational Health.

[B43-behavsci-15-00529] Richard O. C., McMillan-Capehart A., Bhuian S. N., Taylor E. C. (2009). Antecedents and consequences of psychological contracts: Does organizational culture really matter?. Journal of Business Research.

[B44-behavsci-15-00529] Roy S., Mohapatra S. (2023). Exploring the culture–creativity–innovation triad in the handicraft industry using an interpretive approach. Journal of Business Research.

[B45-behavsci-15-00529] Schein E. H. (2010). Organizational culture and leadership.

[B46-behavsci-15-00529] Schimmoeller L. J. (2010). Leadership styles in competing organizational cultures. Leadership Review.

[B47-behavsci-15-00529] Shang D., Wu W., Ji Y. (2025). Understanding employee digital learning engagement and innovative work behavior in hospitality sectors: A machine learning based multistage approach. International Journal of Hospitality Management.

[B48-behavsci-15-00529] Sharma S., Prakash G., Kumar A., Mussada E. K., Antony J., Luthra S. (2021). Analysing the relationship of adaption of green culture, innovation, green performance for achieving sustainability: Mediating role of employee commitment. Journal of Cleaner Production.

[B49-behavsci-15-00529] Song S. I., Kim J. H., Mo Y. M. (2022). The longitudinal study on the impact of innovative organizational culture on organizational commitment. Journal of The Korea Convergence Society.

[B50-behavsci-15-00529] Steenkamp J. B. E. (2001). The role of national culture in international marketing research. International Marketing Review.

[B51-behavsci-15-00529] Su W., Hahn J. (2025). Self-leadership and psychological capital as mediators in the influence of leader motivating language on everyday innovative behavior. International Journal of Business Communication.

[B52-behavsci-15-00529] Tang Y., Shao Y. F., Chen Y. J. (2019). Assessing the mediation mechanism of job satisfaction and organizational commitment on innovative behavior: The perspective of psychological capital. Frontiers in Psychology.

[B53-behavsci-15-00529] Tekleab A. G., Takeuchi R., Taylor M. S. (2005). Extending the chain of relationships among organizational justice, social exchange, and employee reactions: The role of contract violations. Academy of Management Journal.

[B54-behavsci-15-00529] Vuong B. N., Tushar H., Hossain S. F. A. (2022). The effect of social support on job performance through organizational commitment and innovative work behavior: Does innovative climate matter?. Asia-Pacific Journal of Business Administration.

[B55-behavsci-15-00529] Wallace J. C., Butts M. M., Johnson P. D., Stevens F. G., Smith M. B. (2016). A multilevel model of employee innovation: Understanding the effects of regulatory focus, thriving, and employee involvement climate. Journal of Management.

[B56-behavsci-15-00529] Yasin R., Jan G., Huseynova A., Atif M. (2023). Inclusive leadership and turnover intention: The role of follower–leader goal congruence and organizational commitment. Management Decision.

[B57-behavsci-15-00529] Yustrilia I., Sujarwo A., Rofiq A., Ratmawati D., Satriyo B. (2022). The effect of HRM pratices on employee engagement with clan culture as moderating variables. Manajemen Bisnis.

[B58-behavsci-15-00529] Zhan X., Wu J., Jie Y. (2025). How and when psychological safety impacts employee innovation: The roles of thriving at work and regulatory focus. Current Psychology.

[B59-behavsci-15-00529] Zhao L., Bao S., Jolly P. M., Su Y. (2025). Understanding the role of exploitative leadership in inhibiting service innovative behavior: A moderated mediation model. International Journal of Contemporary Hospitality Management.

